# A Neuroscience Perspective of Physical Treatment of Headache and Neck Pain

**DOI:** 10.3389/fneur.2019.00276

**Published:** 2019-03-26

**Authors:** René Castien, Willem De Hertogh

**Affiliations:** ^1^Department of General Practice and Elderly Care Medicine, EMGO Institute for Health and Care Research, VU University Medical Center, Amsterdam, Netherlands; ^2^Amsterdam Movement Sciences, Faculty of Behavioral and Movement Sciences, Vrije Universiteit, Amsterdam, Netherlands; ^3^Department of Rehabilitation Sciences and Physiotherapy, Faculty of Medicine and Health Sciences, University of Antwerp, Antwerpen, Belgium

**Keywords:** physical treatment, headache, neck pain, pain, neurology, clinical reasoning, neurophysiology

## Abstract

The most prevalent primary headaches tension-type headache and migraine are frequently associated with neck pain. A wide variety of treatment options is available for people with headache and neck pain. Some of these interventions are recommended in guidelines on headache: self-management strategies, pharmacological and non-pharmacological interventions. Physical treatment is a frequently applied treatment for headache. Although this treatment for headache is predominantly targeted on the cervical spine, the neurophysiological background of this intervention remains unclear. Recent knowledge from neuroscience will enhance clinical reasoning in physical treatment of headache. Therefore, we summarize the neuro- anatomical and—physiological findings on headache and neck pain from experimental research in both animals and humans. Several neurophysiological models (referred pain, central sensitization) are proposed to understand the co-occurrence of headache and neck pain. This information can be of added value in understanding the use of physical treatment as a treatment option for patients with headache and neck pain.

## Introduction

Headache causes substantial pain and disability in people's daily life and delivers a high burden and cost to society that is estimated only in Europe at 173 billion Euro per year (1). The most prevalent primary headaches worldwide are tension-type headache (TTH) and migraine. These types of headache are frequently associated with neck pain (2, 3). A recent open population study reported a 1-year prevalence of neck pain of 68.4% and more in people with primary headache compared to people without primary headache (85.7 vs. 56.7%; OR 3.0, 95% CI 2.0–4.4). After adjusting for age, gender, education and poor self-rated health, the prevalence of neck pain (56.7%) was still significantly higher in people with only migraine (76.2%), migraine ánd TTH (89.3%), and only TTH (88.4%) in comparison with people without headaches (4). People with headache and neck pain frequently visit health care providers such as medical doctors (general practitioners, neurologists) and physical therapists in their quest for diagnosis and treatment (5). A broad pallet of treatment options is available, including reassurance, self-management strategies, pharmacological, and non-pharmacological treatments. Evidence for the effectiveness of physical therapy for headache is limited (6, 7). Despite this lack of solid scientific back-up, physical therapy is worldwide a frequently used alternative or complementary treatment and included in several clinical guidelines as an alternative treatment option (The European Federation of Neurological Societies (EFNS) guideline, Italian guideline for primary headaches) (5, 8, 9). In daily practice, a combination of treatment options is often used, and the combination of pharmacological (acute and prophylactic drugs) and non-pharmacological (education, physical therapies, exercises, biofeedback) interventions is indeed considered to be an efficient approach in headache disorders (10). Additional research concerning non-pharmacological prophylactic treatment strategies of headache is however urgently needed (11). For disciplines that target the cervical spine in order to decrease headache, it is pivotal for clinical reasoning to understand the neuro-physiological background of headache and neck pain (12). Recently, new insights have emerged on the relation between extracranial input from the (upper) cervical spine and headache from experimental research in both animals and humans (13). This recent information can be of great value to understand and to (re)design physical approaches for different types of headache in combination with neck pain. In this review we first describe the neuro-anatomical and neuro-physiological findings from experimental studies on the trigemino-cervical complex (TCC). We then discuss neurophysiological models to explain the co-occurrence of headache and neck pain such as referred pain and generalized hyperexcitability. We further present the relation of cervical spine dysfunction and headache and research on modulation of nociception at the TCC. Finally, we describe physical treatment as an option to treat headache and neck pain.

## Trigemino-cervical Complex, the Anatomical Basis

**Figure 1:**
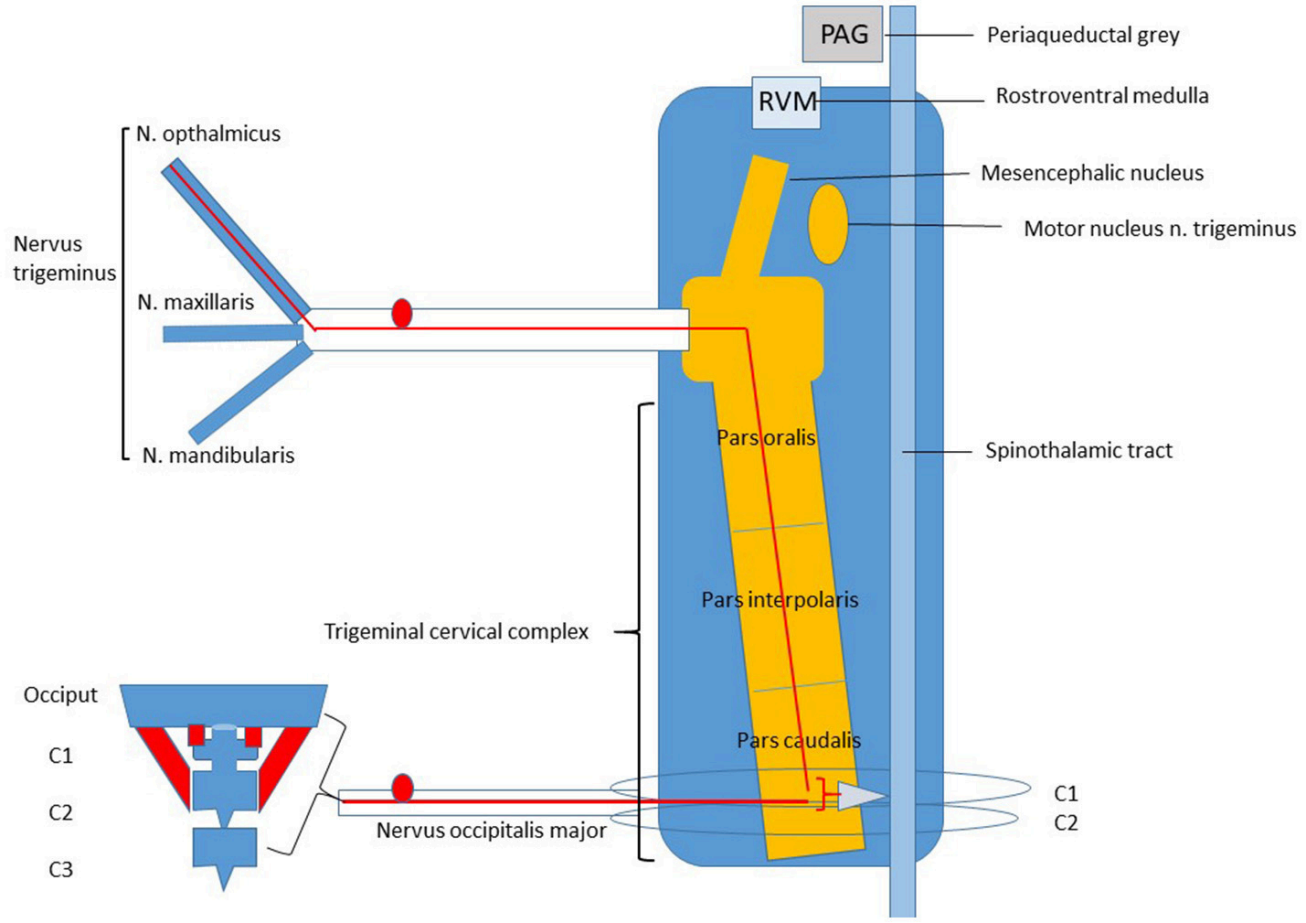
Trigeminal Cervical Complex (TCC).

Experimental research has contributed to further neuro-physiological insights in the relation of headache and neck pain. Knowledge of the neuro-anatomical structures and neural activity within the TCC seems paramount. The frequent co-occurrence of headache and neck pain is attributed to common nociceptive innervation of the head and neck in the dorsal horn C1-2, located in the trigemino-cervical complex. Animal (14, 15) and human (15) anatomical studies have shown that the TCC extends from the medulla (pars oralis and pars interpolaris) to the first and second cervical segments (pars caudalis) (Figure 1). In the TCC, the pars caudalis receive first order nociceptive A^δ^- and C afferent neurons of the ophthalmic nerve to*g*ether with first order A^δ^- and C nociceptive afferent neurons from predominantly the dorsal root C2. These afferent neurons are directly or indirectly connected via wide dynamic range neurons to second-order neurons (16). The ophthalmic nerve delivers nociceptive input via small diameter A^δ^- and C afferent nerve fibers to nociceptive second-order neurons in the superficial and deep layers of the medullary dorsal horn C1 and 2 in the TCC (17, 18). The upper cervical root C2 represents A^δ^- and C nociceptive afferent information of vessels and dura mater of the posterior fossa, and myofascial structures of the upper cervical segments. This nociceptive input from the upper cervical nerve root C2 is well-documented and has a structural overlap with nociceptive nerve endings from the ophthalmic nerve root at the first and second cervical dorsal horn in the TCC (19–27). An extracranial origin of meningeal nociception is suggested by Schueler et al. by demonstrating *in vitro* that collaterals of trigeminal afferents form functional connections between intra- and extracranial tissues in rats and humans. So, information from pericranial muscles can reach the dura mater by ortho- and antidromic conduction through axon collaterals and possibly influence meningeal functions and the generation of headache in humans (28, 29). This finding on collateral afferent connections matches with the anatomical (30) and functional relation (31) of the dura and suboccipital muscles in the upper cervical region in humans. Therefore, the neuro-anatomical connection of ophthalmic and cervical nociceptive afferents on second order neurons at the pars caudalis of the TCC, is pivotal to understand the occurrence of headache and neck pain.

## Referred Pain

The convergence of cervical ánd trigeminal nociceptive small diameter A^δ^- and C fibers on the C1 and C2 dorsal horn provides a neuro-anatomical basis for the clinical phenomenon of referred pain. The co-occurrence of headache and neck pain can be explained by referred pain: pain originating from the neck is perceived as originating from the head and vice versa.

## Evidence From Animal Studies

Animal-experimental neuro-physiological studies recording input of nociceptive afferent fibers at the C1-2 dorsal horn in animals contributed to the understanding of referred pain in both directions, i.e., from the neck to the head (20) and from the head to the neck (21). Vernon et al. described the increased activity in C1/C2 dorsal horns in rats after injection of inflammatory mustard oil in deep paraspinal tissues at the level of the left C1-C2 joint. Activation of trigeminal afferents of the supratentorial dura mater by mustard oil (MO) showed an enlargement of cervical cutaneous mechanoreceptive fields together with a significant (*p* < 0.001) increase in the excitability to electrical stimulation of the greater occipital nerve in C-fiber responses (21). Unilateral electric stimulation of the greater occipital nerve in cats increased metabolic activity in the dorsal horn C1 and C2. Stimulation of trigeminally-innervated structures showed a similar distribution to the trigeminal nucleus caudalis (32). Based on these findings, the well-recognized clinical phenomenon of head pain that is perceived frontal and occipital and in the upper neck may be the result of overlap of nociceptive information at the level of second order neurons.

Headache during a migraine attack seems to be primarily based on activation of the trigeminovascular pathways by increased visceral nociceptive A^δ^- and C fibers input of the dura and intracranial vessels on the TCC. This input is frequently restricted to the territory of the ophthalmic nerve, but may extend as pain to the occipital region of the head which is innervated by the greater occipital nerve C2 (33). These results indicate that headache as well as neck pain can be perceived as referred pain.

## Evidence From Human Studies

Clinical evidence of referred pain based on convergence of cervical- and ophthalmic nociceptive A^δ^- and C afferent input originating from different structures has been observed in human studies. Clinical observations have shown that intracranial nociceptive input of arteries, but also extracranial nociceptive input originating from the vertebral artery is able to provoke painful sensations in the area of the forehead (34, 35). Provocation of headache by applying experimental nociceptive stimuli to upper cervical structures has been reported in several studies. Injection of saline in the neck and suboccipital region (36), sterile water (37) and low-frequency nerve stimulation (38) over the upper cervical dorsal roots have shown to provoke headache. In a narrative review on the diagnosis and treatment of cervicogenic headache, Bogduk has described several experimental studies on humans reporting referred pain patterns on the head caused by stimulation of nociceptive afferent input from myofascial structures of the upper cervical spine (39). In 23 out of 32 patients with cervicogenic headache the pain in the head was relieved completely after a diagnostic anesthetic block at the lateral atlanto-axial joints (C1-2) (40). Mechanical nociceptive afferent stimuli -by giving a firm pressure to myofascial structures of upper cervical segments (C0-3)- also provoke the patient's typical headache in patients with cervicogenic headache (41), TTH, and migraine (42, 43). Extensive research is available on trigger points in cervical and suboccipital muscles eliciting headache (44). In summary, convergence of cervical and trigeminal nociceptive afferents on second order neurons at the TCC can cause headache as referred pain via stimulation of cervical nociceptive input of the upper cervical segments by administration of fluid-irritants or mechanical pressure.

## Generalized Hyperexcitability

Hyperexcitability of second order neurons in the TCC as a result of a continuous increased peripheral somatic and vascular nociceptive activity (45–48), a decrease of supraspinal inhibition (49) or a combination of both mechanisms can cause headache (50, 51). Activation of the trigeminovascular pathways increased by vascular nociceptive A^δ^- and C fibers input of the dura and intracranial vessels on the TCC seems to be typical for migraine (47). Still, at present there is an ongoing debate what is causing the hyperexcitability of second order neurons in the TCC during migraine. Levy et al noticed that sensory innervation of the cranial meninges and immune and vascular cells may have a major role, but evidence for neurogenic inflammation during migraine and its contribution to meningeal nociception is limited (52). Prolonged or ongoing peripheral nociceptive input via trigger points in pericranial or cervical myofascial structures may contribute to hyperexcitability of second-order neurons at the C1 and C2 dorsal horn of the TCC in TTH, but evidence for this hypothesis is limited (53). Hyperexcitability of nociceptive second order neurons in the dorsal horn of C1-2 can also be caused by a decrease of endogeneous driven supraspinal descending inhibition of the periaqueductal gray (PAG), nucleus raphe magnus, or rostroventral medulla. This can lead to clinical signs such as hypersensitivity, allodynia and reduced pain thresholds in the cranio-cervical region and even in extra- cephalic regions. In patients with chronic TTH, but not with episodic TTH, most studies report lower pressure, thermal and electrical pain thresholds in the cephalic region (54). In patients with migraine pain threshold to pressure, cold and heat stimuli in the cephalic region are found to be lower during the ictal phase than during the interictal phase of migraine or healthy controls (55). For pain pressure thresholds in the cranio-cervical region a significant decrease is described in research on patients with migraine and CTTH compared to healthy controls (56). The interaction between supraspinal descending inhibitory systems and peripheral nociceptive input in the TCC seems to be a prerequisite for the characteristics as well as in the development of episodic to chronic headache syndromes (57). Thus, trigger points or tender, painful myofascial structures at the upper cervical segments in headache patients can either emerge or be a source of hyperexcitability of second-order neurons C1-C2.

## Cervical Musculoskeletal Dysfunctions in Headache

Cervical musculoskeletal dysfunctions of joints and muscles have been observed in patients with migraine, TTH and cervicogenic headache (58–62). In the context of the neurophysiological interconnection between the dorsal root of C2 (greater occipital nerve) and the TCC, it may be not surprising that in participants with headache most cervical musculoskeletal dysfunctions are present in the upper cervical spine. Palpation of trigger points in suboccipital muscles and trapezius (63–66), restricted motion of the cervical segments C0-3 (43, 67), and stress on joints in the upper cervical spine (41, 42) are related to different types of headache. Although there seems to be a relation between (upper) cervical musculoskeletal dysfunctions and headache, these are documented in studies with a case–control design. Thus, no causal relation can be determined, nor solid conclusions can be drawn on this relation.

## Modulation of Nociception at the TCC: Evidence From Animal Studies

Evidence is emerging that addressing the cervical spine can modulate pain at the TCC. Nöbel et al. reported that injection of a nociceptive stimulant (α,β-meATP) into the temporal muscle in rats induces ongoing activity of spinal trigeminal neurons with meningeal receptive fields. In the same study local anesthesia of single neck muscles, but not of the musculus temporalis, shows a significant decrease of the provoked central trigeminal activity (68). This supports the modulation of pain in the TCC by reduction of peripheral cervical muscular nociceptive afferent input. Supraspinal diffuse noxious inhibitory control (DNIC) on convergent neurons in the trigeminal nucleus caudalis in rats can be initiated by activation of A^δ^- and C fibers. Villaneuva et al. and Bouhassira et al. demonstrated that induced activity of convergent neurons in the trigeminal nucleus caudalis was decreased up to 80% by activation of A^δ^- and C fibers (69, 70). Afferent A^δ^- and C input originating from the neck is not restricted to the TCC. Local administration of nerve growth factor into semispinal neck muscles in anesthetized mice shows not only stronger Fos immunoreactivity in the superficial layers I and II of the of cervical spinal dorsal horns C1, C2, and C3, but also in supraspinal structures such as the PAG and the medullary lateral reticular nucleus (71–76). Nearly 50% of all ventro-lateral PAG-projecting spinal neurons were found in the upper cervical segments and these segments are thereby potentially an important source to activate the ventrolateral PAG (71, 77). Activation of the ventrolateral PAG by deep somatic (deep neck muscles) and visceral pain not only leads to a resting state, but also to inhibition of trigeminal afferents (76, 78). The participation of this phenomenon in inhibition of trigeminal afferents is proposed (79, 80).

## Modulation of Nociception at the TCC: Evidence From Human Studies

In a clinical study, Busch et al established modulation of nociception at the TCC by detecting a decrease of R2 response areas (AUC) and significantly increased R2 latencies of the nociceptive blink reflex only at the side of an anesthetic unilateral nerve blockade of the greater occipital nerve with prilocaine in healthy persons. These findings not only confirmed previous results related to anatomical and functional convergence of trigeminal and cervical afferent pathways, but also suggested that modulation hereof could be beneficial in treatment of primary headache disorders (81). In patients with headache, blocking afferent nociceptive input by anesthesia of the GON (82, 83) or in the facet joint C1-2 (40, 84) has proven to be effective in reducing headache. Piovesan et al. described the decrease of headache in a patient with migraine after light massage of the greater occipital nerve (85). Another clinical study by Watson and Drummond (42) reported the provocation as well as the resolution of headache in migraine patients with sustained manual pressure in the suboccipital region. The referred pain during the provocation test was decreased in parallel with a change in the trigeminal nociceptive blink reflex. This finding supposes the previously proposed model that stimulation of myofascial A^δ^- and C fibers by manual pressure can activate the supraspinal DNIC system that acts specifically on spinal wide-dynamic-range (WDR) neurons and is able to modulate nociception at the TCC (69, 86).

## Physical Treatment of Headache and Neck Pain

The neuro-anatomical and—physiological relation between brainstem nuclei, the (upper) neck and trigeminal nerve has to be incorporated in development of physical treatment for headache targeted at the cervical spine, especially the upper cervical region. According to the ‘gate-control' hypothesis, the relative high amount of proprioceptive afferent muscular input of upper cervical segments (87) to the central nervous system may alter nociceptive A^δ^- and C fibers afferent input. Stimulation of proprioceptive input by active exercises for neck muscles may decrease the excitability of second order neurons at the TCC (11) and activation of the supraspinal DNIC system by stimulation of myofascial A^δ^- and C fibers by manual pressure techniques at the upper cervical spine can be of added value (42). The importance of an active treatment of neck muscles is supported by the findings of a systematic review of Varatharajan et al. stating that an active physical treatment including exercises shows promising results on reduction of headache associated with neck pain (7).

## Discussion

In the last decades experimental research in both animals and humans on neuro-anatomy and neuro-physiology has contributed to understand the co-occurrence of headache and neck pain. Based on this information we further present a neuro-physiological background for physical treatment of headache and neck pain. Studies have gain new insights on the neuro-anatomical and neuro-physiological relation between headache and neck pain, but also raise questions if and how this relation can be influenced by physical treatment. Headache (migraine, tension-type headache, cervicogenic headache), neck pain, and cervical musculoskeletal dysfunctions seem to be related in case-control studies, although the strength, significance and explanation of this relation varies per type of headache.

Clinicians have to consider, by sound clinical reasoning, whether cervical musculoskeletal dysfunctions are related to the patient's headache and which neurophysiological mechanisms could be involved. Therefore, we support the recommendation to classify headache according to the ICHD III criteria and to determine cervical musculoskeletal dysfunctions in patients with migraine, tension-type headache and cervicogenic headache (88). Additionally, tests on pain sensitivity can be included to understand the underlying pathophysiological mechanism. In their clinical judgement, clinicians have to consider all collected patient data: headache symptoms and neck pain, related cervical musculoskeletal dysfunction, tests on pain sensitivity in the cervico-cephalic and extra-cervico-cephalic regions (pressure pain thresholds) and reproduction of headache by pressure or stretch on musculoskeletal structures (43). To understand underlying neurophysiological mechanisms (local nociceptive provocation, referred pain, generalized hyperexcitability) remains challenging, but is necessary to identify patients who may benefit of treatment of the neck (89). The presented neurophysiological knowledge in this paper can be helpful to guide clinicians in this clinical reasoning process.

It is a great challenge for clinicians and researchers to develop effective treatment strategies for headache targeted on modulation of cervical afferent input in order to decrease the excitability of first- to second order neurons at the level of the TCC. Experimental studies of the neurophysiological effect of physical treatment and randomized clinical trial on this topic are scarce and urgently warranted. Meanwhile, there is no standard recipe for physical treatment on the neck for different types of headache. But clinicians may be encouraged by recent evidence and new insights on headache and neck pain and may use this knowledge in clinical reasoning to provide a tailored and evidence based neuro-physiological approach for patients with headache and neck pain.

## Author Contributions

RC and WD: concept development and writing of the manuscript; Both authors approved the final version.

### Conflict of Interest Statement

The authors declare that the research was conducted in the absence of any commercial or financial relationships that could be construed as a potential conflict of interest.
